# A Phase 1/2A trial of idroxioleic acid: first-in-class sphingolipid regulator and glioma cell autophagy inducer with antitumor activity in refractory glioma

**DOI:** 10.1038/s41416-023-02356-1

**Published:** 2023-07-24

**Authors:** Juanita Lopez, Julia Lai-Kwon, Rhoda Molife, Liam Welsh, Nina Tunariu, Desamparados Roda, Paula Fernández-García, Victoria Lladó, Adrian G. McNicholl, Catalina A. Rosselló, Richard J. Taylor, Analía Azaro, Jordi Rodón, Julieann Sludden, Gareth J. Veal, Ruth Plummer, Ander Urruticoechea, Ainhara Lahuerta, Karmele Mujika, Pablo V. Escribá

**Affiliations:** 1 The Royal Marsden Hospital and the Institute of Cancer Research, Sutton, UK; 2 Laminar Pharmaceuticals, Palma de Mallorca, Spain; 3 Hospital Vall d’Hebrón, Barcelona, Spain; 4 Northern Centre for Cancer Care, Newcastle upon Tyne, UK; 5 Gipuzkoa Cancer Unit, OSID-Onkologikoa, San Sebastián, Spain

**Keywords:** Cancer therapy, Phase I trials

## Abstract

**Background:**

The first-in-class brain-penetrating synthetic hydroxylated lipid idroxioleic acid (2-OHOA; sodium 2-hydroxyoleate), activates sphingomyelin synthase expression and regulates membrane-lipid composition and mitochondrial energy production, inducing cancer cell autophagy. We report the findings of a multicentric first-in-human Phase 1/2A trial (NCT01792310) of 2-OHOA, identifying the maximum tolerated dose (MTD) and assessing safety and preliminary efficacy.

**Methods:**

We performed an open-label, non-randomised trial to evaluate the safety, tolerability, pharmacokinetics, pharmacodynamics and anti-tumour activity of daily oral treatment with 2-OHOA monotherapy (BID/TID) in 54 patients with glioma and other advanced solid tumours. A dose-escalation phase using a standard 3 + 3 design was performed to determine safety and tolerability. This was followed by two expansion cohorts at the MTD to determine the recommended Phase-2 dose (RP2D).

**Results:**

In total, 32 recurrent patients were enrolled in the dose-escalation phase (500–16,000 mg/daily). 2-OHOA was rapidly absorbed with dose-proportional exposure. Treatment was well-tolerated overall, with reversible grade 1–2 nausea, vomiting, and diarrhoea as the most common treatment-related adverse events (AEs). Four patients had gastrointestinal dose-limiting toxicities (DLTs) of nausea, vomiting, diarrhoea (three patients at 16,000 mg and one patient at 12,000 mg), establishing an RP2D at 12,000 mg/daily. Potential activity was seen in patients with recurrent high-grade gliomas (HGG). Of the 21 patients with HGG treated across the dose escalation and expansion, 5 (24%) had the clinical benefit (RANO CR, PR and SD >6 cycles) with one exceptional response lasting >2.5 years.

**Conclusions:**

2-OHOA demonstrated a good safety profile and encouraging activity in this difficult-to-treat malignant brain-tumour patient population, placing it as an ideal potential candidate for the treatment of glioma and other solid tumour malignancies.

**Clinical trial registration:**

EudraCT registration number: 2012-001527-13; Clinicaltrials.gov registration number: NCT01792310.

## Introduction

Gliomas are the most common malignant brain tumour in adults [1]. Standard of care consists of surgical resection, adjuvant radiotherapy plus concomitantly administered temozolomide (TMZ) in patients with good Karnofsky performance status [2]. Prognosis is poor with median overall survival for WHO Grade-IV (according to the WHO 2017 classification criteria) glioblastoma (GBM) to be 14–18 months at best [3–5], with no significant improvements in outcomes since the introduction of radiotherapy-TMZ in 2005.

**Fig. 1:**
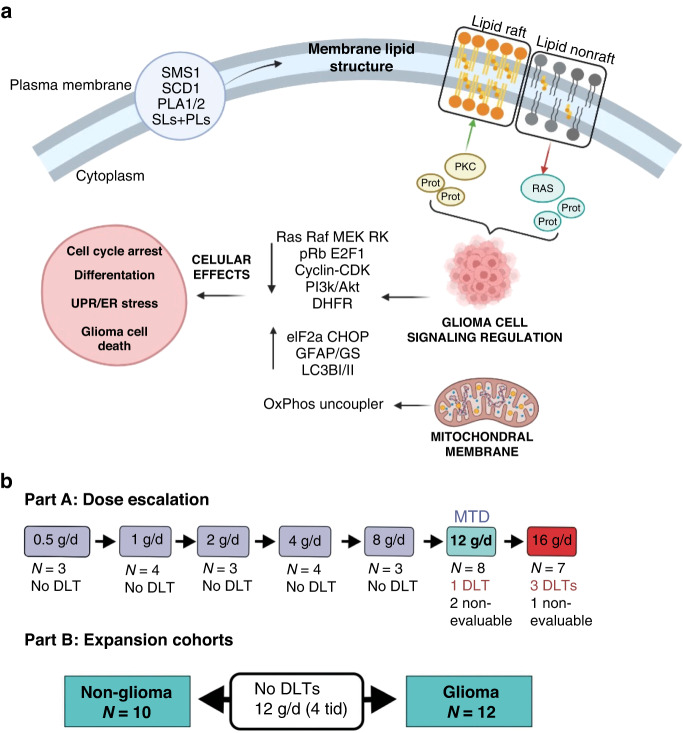
Schemes of cell signalling regulation by 2-OHOA treatment and clinical trial. **a** Mechanism of action of 2-OHOA. (1) 2-OHOA regulates the cell membrane composition by both direct incorporation into the lipid bilayer and regulation of important lipid metabolism enzymes, such as SMS-1, stearyl CoA desaturase 1 (SCD1), phospholipases 1 and 2 (PLA1/2), which regulate the levels and types of sphingolipids (SLs) and phospholipids (PLs). (2) Among other structural effects on the lipid bilayer, these changes in lipid composition regulate the raft-to-nonraft lipid balance, which causes translocation of numerous proteins from or to the membrane (e.g., PKC and Ras). (3) The presence or absence of peripheral membrane signalling proteins triggers or hampers, respectively, important signalling cascades. (4) These signalling cascades control glioma cell’s physiology, inducing unfolded protein response (UPR), endoplasmic reticulum (ER) stress, cell cycle arrest, cell differentiation, oxidative phosphorylation uncoupling, and eventually programmed cell death (autophagy). **b** Clinical trial scheme: Dose escalation had a 3 + 3 scheme. All cohorts received the indicated daily doses in two oral administrations, except those cohorts receiving 12,000 mg/d, which were administered TID (4000 mg per dose).

Cell membrane-lipid composition and structure are markedly altered in cancer cells with the resulting impact on intracellular oncogenic signalling [6–9]. Sphingolipids regulate key cancer signalling transduction networks and play critical roles in oncogenesis by modulation of cell cycle, apoptosis, angiogenesis, endoplasmic reticulum (ER)-stress and inflammation [10–12]. Sphingolipids are crucial for cell membranes, with sphingomyelin synthase (SMS) as a key player (Fig. 1). SMS-1 is the main isoform and is present at significantly lower levels in cancer cell membranes, including GBM [13]. In addition, low SMS-1 expression in gliomas is prognostic for poorer outcomes [13, 14].

2-OHOA, granted orphan designation for the treatment of glioma in both the European Union (EU) and the United States (USA), is a novel oral synthetic hydroxylated fatty acid derived from oleic acid acting via a novel technology, melitherapy (membrane-lipid therapy), regulating the membrane-lipid composition and structure in glioma and other tumours through SMS-1 expression activation [6, 15, 16]. 2-OHOA modulates multiple signal-transduction pathways including RAS/RAF/mitogen-activated protein kinase, phosphatidylinositol 3-kinase/Akt/PTEN/mTOR and induces ER-stress followed by autophagic cell death both in cellula and in vivo human glioma mouse models [6, 7, 17–19] (Fig. 1). Importantly, 2-OHOA can penetrate the blood–brain–barrier with activity in orthotopic glioma models, both as single agent and in combination with TMZ [6], where is preferentially taken up by glioma cells [20]. In addition, 2-OHOA regulates mitochondrial membrane lipids inducing uncoupling of oxidative phosphorylation in human glioma cells but not in normal cells [21]. Based on these data, we conducted a first-in-human, first-in-class study exploring 2-OHOA safety and tolerability in patients with solid tumours and gliomas.

## Patients and methods

Study protocol MIN-001-1203 was a first-in-human, multicentric, open-label Phase I/IIA study in patients with recurrent advanced solid tumours and high-grade gliomas (HGGs; ClinicalTrials.gov identifier: NCT01792310). All enrolled patients provided written informed consent. The study was performed in accordance with the Declaration of Helsinki and the International Council for Harmonisation Guidelines on Good Clinical Practice and approved by the institutional review board or ethics committees at participating institutions. The study was designed by the sponsor (Laminar Pharmaceuticals) in collaboration with the study investigators (Supplemental Text 1).

The primary objectives were to determine the safety and tolerability of 2-OHOA, to describe the maximum tolerated dose (MTD), dose-limiting toxicities (DLTs), and to identify the recommended Phase-2 dose (RP2D; Supplemental Text 2). Secondary objectives included the characterisation of the single and steady-state pharmacokinetic (PK) profiles of 2-OHOA on a continuous daily dosing schedule, and to assess its preliminary antitumor efficacy.

Adverse events (AEs) were evaluated according to National Cancer Institute–Common Terminology Criteria for AEs (NCI-CTCAE), version 4.03. Disease was documented by computed tomography (CT) or magnetic resonance imaging (MRI). Tumours were evaluated at baseline and after every two cycles until disease progression according to RANO criteria for gliomas, or RECIST 1.1 for solid tumours.

Patients of age ≥18 years with histologically or cytologically-confirmed advanced solid tumours, including HGGs refractory to standard-of-care treatment, or for which there was no standard treatment, were included (Table 1 and Supplemental Table 1). In the glioma cohort, patients with Grade-III or -IV (as classified when the trial was performed, all of them are considered high-grade glioma) malignant glioma that had recurred or progressed after first- or second-line standard-of-care treatment were included, with progressive disease defined according to RANO criteria. Previous lines of treatment received by the patients are listed in Supplemental Table 1, and the molecular features of glioma patients are indicated in Supplemental Table 2.

**Table 1:** Patient demographics and baseline characteristics and overview of the study population.

All patients	
Median age, years (range)	59.0 (19–78)
Gender, *n* (%)	
Male	32 (59.3%)
Female	22 (40.7%)
Median number of prior lines of therapy	4
ECOG PS (%)	
0	8 (16.6%)
1	44 (81.48%)
2	3 (5.5%)
Primary tumour site (*n*)	
GI	15
Gynae	3
Lung	3
Glioma	27
Other solid tumours	6
GLIOMA patients	
Median age, years	51.9
Gender, *n* (%)	
Male	16 (59.3%)
Female	11 (40.7%)
Histological classification (%)	
WHO Grade-IV GBM (current stage 4)	18 (66.7%)
High=grade glioma, other	9 (33.3%)

Study population		Included in the analysis, *N* (%)		
		Glioma (*N* = 27)	Other solid tumour (*N* = 27)	Total (*N* = 54)
SAF population		27 (100%)	27 (100%)	54 (100%)
ITT Population	Overall	27 (100%)	27 (100%)	54 (100%)
	PFS	27 (100%)	27 (100%)	54 (100%)
	Tumour response	21 (77.8%)	24 (88.8%)	45 (83.3%)
PPS population	Overall	14 (51.9%)	19 (70.4%)	33 (61.1%)
	PFS	14 (51.9%)	19 (70.4%)	33 (61.1%)
	Tumour response	13 (41.8%)	19 (70.4%)	32 (59.3%)
PK population		27 (100%)	27 (100%)	54 (100%)

*ITT* intention to treat, *PK* pharmacokinetic, *PPS* per-protocol set, *SAF* safety.

Key exclusion criteria included anticancer therapy within 4 weeks prior to treatment initiation (6 weeks for mitomycin and nitrosoureas and 2 weeks for palliative radiotherapy and surgery); unresolved grade >1 NCI-CTCAE from prior anticancer therapy; gastrointestinal dysfunction that could alter drug absorption; a history of hyperlipidemia and/or need for lipid-lowering therapy; significant uncontrolled cardiovascular disease; and recent intracranial or intratumor haemorrhage on CT or MRI.

2-OHOA was administered as a dry powder reconstituted as an oral suspension 30 min to 2 h after food in 21-day cycles which may be repeated continuously without therapy interruption, until any criterion for discontinuation (clinical or radiological progression of disease, clinically unacceptable toxicity, or another “general” discontinuation criterion) is met. The starting dose was 250 mg BID. Higher doses could be administered t.i.d. A standard “3 + 3” dose-escalation design was used with seven cohorts (500–16,000 mg total daily dose, Fig. 1). Three patients were enrolled per dose level and observed for any DLT during the first cycle of treatment. Dosing proceeded to higher dose levels if no DLTs were observed in any patient. An additional three patients were enrolled at dose levels where one out of three patients experienced a DLT. Within each cohort, patients were entered ≥1 week between the first and subsequent patients.

To be evaluable for dose-escalation decisions, patients either had to have taken ≥80% of the study drug or have experienced a DLT during the DLT observation period.

Dose cohort escalation decisions were also based on a clinical review of all relevant available data from contemporaneous and previous dose cohorts. The maximum administered dose was defined as the dose level at which a DLT was observed during treatment cycle 1 in ≥33% of evaluable patients, and the MTD was defined as the highest dose level below the former.

Treatment was continued until clinical or radiological disease progression, unacceptable toxicity, withdrawal of consent or investigator decision. Treatment schedule modifications, dose delays of up to 14 days and up to two dose reductions were permitted for toxicity. Intra-patient dose escalations were permitted if the patient had derived clinical benefit on their initial or current dose level, at the investigator’s discretion and in consultation with the Medical Monitor.

PKs were assessed using a fully validated LCMS/MS to measure 2-OHOA blood concentrations with a linear range of 25–1500 ng/ml. PK profiles (pre-dose, and 1-, 2-, 4-, 6- and 8-h post-dose) were determined on days 1 and 21 of cycle 1 of treatment. PK data were analysed by noncompartmental methods using WinNonlin Version 6.4. Maximum concentration achieved (*C*_max_), area under the plasma concentration-time curve (AUC), volume of distribution (*V*_d_) and clearance (CL) were calculated. In the dose-escalation cohorts, trough levels were also measured pre-dose on days 8 and 15. In the expanded safety cohort, only the day 1 PK profile was measured. A final PK sample was taken at the end of study visit.

The effect of food on the PK of 2-OHOA was also assessed in the first four dose cohorts. The 8-h PK profile on day 1 was performed 30 min after the investigational site’s standard breakfast or 500 mL Ensure® and a further 48-h fasting PK profile was obtained after a single run-in dose of 2-OHOA on day -7 in these patients. Standard noncompartmental pharmacokinetic parameters were estimated after single and multiple dosing for fasting and non-fasting conditions. Accumulation parameters were calculated by comparing the day 1 and day 21 PK profiles.

The planned maximum sample size was determined by modelling dose escalation stopping clinical criteria, with additional patients in the two expanded safety cohorts (12 with malignant HGG, 10 with other advanced solid tumours: Fig. 1).

Patient Study Population considered for different analysis is indicated in Table 1. Five analysis populations were defined to analyse the study data:

- Safety Analysis (SAF) Population: Patients who received at least one dose of 2-OHOA.
- Intention-to-Treat (ITT) Population: All patients in the SAF who have baseline and post-treatment tumour response data or progression-free survival (PFS) data.
- Per-protocol Set (PPS) population: All patients in the ITT population who fully complied with the inclusion and exclusion criteria, did not take prohibited medications and did not miss >20% of doses and had no major protocol deviations.
- PK Population: Patients who received 2-OHOA and provided at least one evaluable pre-dose and post-dose PK blood sample and with no major protocol deviations that impacted the PK data. Patients who had a fasting run-in dose at day -7 to -5, had their Cycle 1 day 1 dose fed, and gave at least one pre-dose and post-dose sample on one of these days, were included in fasted/fed sub-analysis.
- The efficacy analysis population included all patients who received at least ≥80% of doses administered in cycle 1 and who underwent at least one on-study tumour assessment (Table 1).

Responses were summarised descriptively by frequency distributions. Median progression-free survival (PFS) was estimated by Kaplan–Meier analysis. PFS at 6 months (i.e., percentage of patients alive and progression-free at 180 days after treatment initiation) was also determined. All analyses were performed using SAS statistical software version 9.4.

PK parameters were summarised using descriptive statistics. Individual and mean concentration versus time profiles were presented on a linear and logarithmic scale. A Power Model was used to test dose proportionality.

## Results

Fifty-four patients were enrolled and treated at 5 investigational sites in the United Kingdom and Spain. Study design included two phases: Dose-escalation phase (DE) and Expansion Phase (EP) at the Maximum tolerated dose determined at the DE. No patient withdrew due to an AE or a serious AE (SAE) in either the dose escalation or expansion phases of the study. Thirty-two patients (15 with HGG, 17 with other advanced solid tumours) were enrolled in the DE of the study. Twenty-five (78.1%) patients were followed up until disease progression, 5 (15.6%) patients withdrew consent, and 2 (6.3%) discontinued treatment due to an investigator decision. Twenty-two patients (12 with HGG, 10 with other advanced solid tumours) were enrolled in the expanded safety cohorts, and all patients received study drug. In the expansion cohort, only 1 patient (5.4%) discontinued due to non-compliance with study drug, while the remaining 31 (95.5%) discontinued due to disease progression.

Patient baseline characteristics are listed in Table 1 and Supplemental Table 1. All patients had received prior systemic therapy, with approximately half receiving 3–5 lines of therapy (Supplemental Table 1). All patients with HGG had completed chemo-radiation and adjuvant TMZ, and 13% (7/54) had also received prior anti-angiogenic therapy. All patients had active progression of the tumour on study entry and an expectancy of life over 3 months.

Thirty-two patients were included in the DE at dose levels ranging from 500 to 16,000 mg (daily, p.o.: Fig. 1). Twenty-two patients in the EP received the MTD of 12,000 mg daily. The mean cumulative dose was 318,571.4 mg (1143–2,912,571 mg), and the median duration of treatment was 41 (2-989) days. Twelve (22.2%) patients had <1 cycle, 22 (40.7%) had 1 cycle, 13 (24.1%) had 2–5 cycles, 4 (7.4%) patients had 6–10 cycles and 3 (5.5%) had >10 cycles. Fifteen (27.8%) patients required a dose interruption, 1 patient (16,000 mg dose cohort) received a dose reduction to 12,000 mg daily and 2 patients (500 mg and 1000 mg dose cohorts) received an intra-patient dose escalation.

Treatment compliance was defined as taking ≥80% of the intended dose of 2-OHOA. During cycle 1, 38 (70.4%) patients were deemed treatment compliant, including 24 (75%) patients during the dose-escalation phase and 14 (63.6%) patients in the expanded safety cohorts. Across all treatment cycles, 33 (61.1%) patients were treatment compliant, including 22 (60.8%) during the dose-escalation phase and 11 (50%) in the expanded safety cohorts.

Fifty-four patients were included in the safety analysis. All patients experienced at least 1 treatment-emergent AEs (TEAE) during the study. The most common TEAEs were diarrhoea (31 [57.4%]), nausea (23 [42.6%]) and vomiting (18 [33.3%]) (Table 2). Other events reported by ≥20% of the patients were ALT increase (12 [22.2%]), constipation (11 [20.4%]) and decreased appetite (11 [20.4%]). ≥Grade-3 TEAEs occurred in 25 (46.3%) patients. Three (5.6%) patients had a Grade-4 TEAE, none of which was assessed as study-drug-related.

**Table 2:** Treatment-emergent adverse events occurring in >10% of patients in total (safety analysis set) by CTCAE grade.

AE	Dose escalation							Safety Expansion			
	Cohort 1—250 mg/d BID	Cohort 2—500 mg/d BID	Cohort 3—1000 mg/d BID	Cohort 4—2000 mg/d BID	Cohort 5—4000 mg/d TID	Cohort 6—8000 mg/d BID	Cohort 7—16,000 mg/d BID	Cohort 30—4000 mg/d t.i.d.	Cohort 40—4000 mg/d t.i.d.	Total	
Number of patients (% of total)	3 (5.5%)	4 (7.4%)	3 (5.5%)	4 (7.4%)	3 (5.5%)	8 (14.8%)	7 (13%)	12 (22.2%)	10 (18.5%)	54 (100%)	
										G1-2	G3-4
Diarrhoea		2 (50.0%)	1 (33.3%)	2 (50.0%)	2 (66.7%)	4 (50.0%)	6 (85.7%)	7 (58.3%)	4 (40.0%)	26 (48.1%)	2 (3.7%)
Nausea		2 (50.0%)		1 (25%)	2 (66.7%)	2 (25.0%)	5 (71.4%)	2 (16.7%)	4 (40.0%)	17 (31.5%)	1 (1.9%)
Vomiting				1 (25.0%)	2 (66.7%)	3 (37.5%)	6 (85.7%)		3 (30.0%)	16 (29.6%)	1 (1.9%)
Decreased appetite					2 (66.7%)	1 (12.5%)	3 (42.9%)		2 (20.0%)	8 (14.8%)	0 (0.0%)
Alanine aminotransferase increased		1 (25.0%)		1 (25.0%)		1 (12.5%)		3 (25.0%)		6 (11.1%)	0 (0.0%)
Fatigue							2 (28.6%)	2 (16.7%)	2 (20.0%)	6 (11.1%)	0 (0.0%)
Serious AEs											

Forty-two (77.8%) patients experienced at least one study-drug-related TEAE. The most frequent study-drug-related TEAEs were diarrhoea (28 [51.8%]), nausea (18 [33.3%]), and vomiting (17 [31.5%]) (Table 2). The incidence of these events appeared to be dose-related, with all events being absent at 500 mg per day and the highest incidence at 16,000 mg per day. Four (12.5%) patients had a DLT (diarrhoea, drug intolerance, vomiting, abdominal pain) of Grade 2 or Grade 3 following the two highest doses of 2-OHOA in the dose-escalation phase (1 patient at 12,000 mg daily and 3 patients at 16,000 mg daily). Two (3.7%) patients had a TEAE leading to dose reduction during the dose-escalation phase. Eleven (20.4%) patients had a TEAE leading to discontinuation of study drug. However, only 1 event was assessed as study-drug-related (one patient on the 12,000 mg daily dose-escalation cohort experienced grade 1 diarrhoea). There were no study-drug-related SAEs nor study-drug-related deaths.

Increases in alkaline phosphatase, ALT, AST, urea and total bilirubin to above normal range were noted in 10–50% of patients, but there was no apparent dose effect and shifts were typical of a patient’s disease. There was no apparent dose effect on fasting blood lipid parameters or serum amylase. There were no clinically significant study-drug-related ECGs changes.

**Fig. 2:**
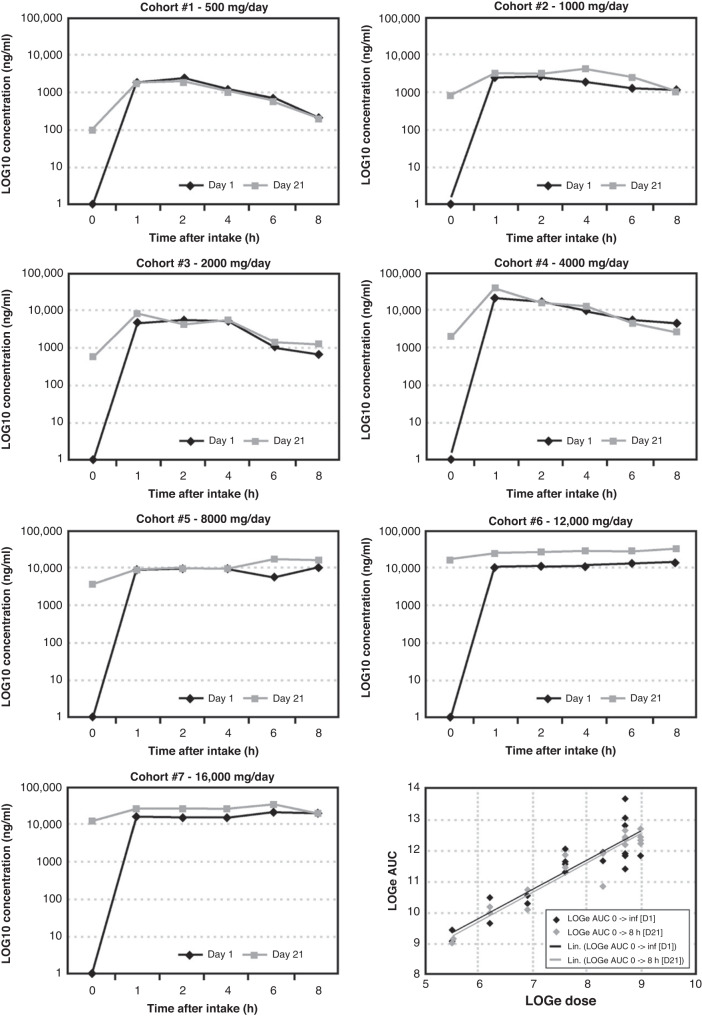
Pharmacokinetic profiles of 2-OHOA at different doses. The graphs represent log [2-OHOA] (ng/ml) in serum of patients after single dose (day 1, black) and steady state (day 21, black) at doses ranging from 500 mg/d to 16,000 mg/d (p.o.). Bottom/right panel: Linear relationship between the extent of systemic exposure (area under the curve, AUC) and dose following single (day 1, D1, black) and repeat BID (day 21, D21, grey) administration of 2-OHOA.

PK findings showed that 2-OHOA was quantifiable across the dose range investigated (Fig. 2). Food intake did not alter oral 2-OHOA bioavailability. However, food caused a statistically significant delay in *T*_max_ (*T*_max_ fasted: 1–1.3 h, *T*_max_ fed: 1.6–3.2 h, *P* = 0.0036). The power model showed dose proportionality in terms of AUC and *C*_max_ after single (250–4000 mg) and multiple (500–16,000 mg) dosing. Systemic exposure (*C*_max_ and AUC) increased from the first dose on day 1 to last dose on day 21 following continuous dosing. Accumulation was observed from 1000 mg per day. The accumulation ratio after continuous twice daily and three times daily dosing was ~1.5 and 2, respectively. The terminal elimination half-life could not be accurately determined due to the lack of sampling points at this phase of the plasma concentration-time curve. However, the effective half-life was between 8 and 9 h based on the accumulation ratio.

**Fig. 3:**
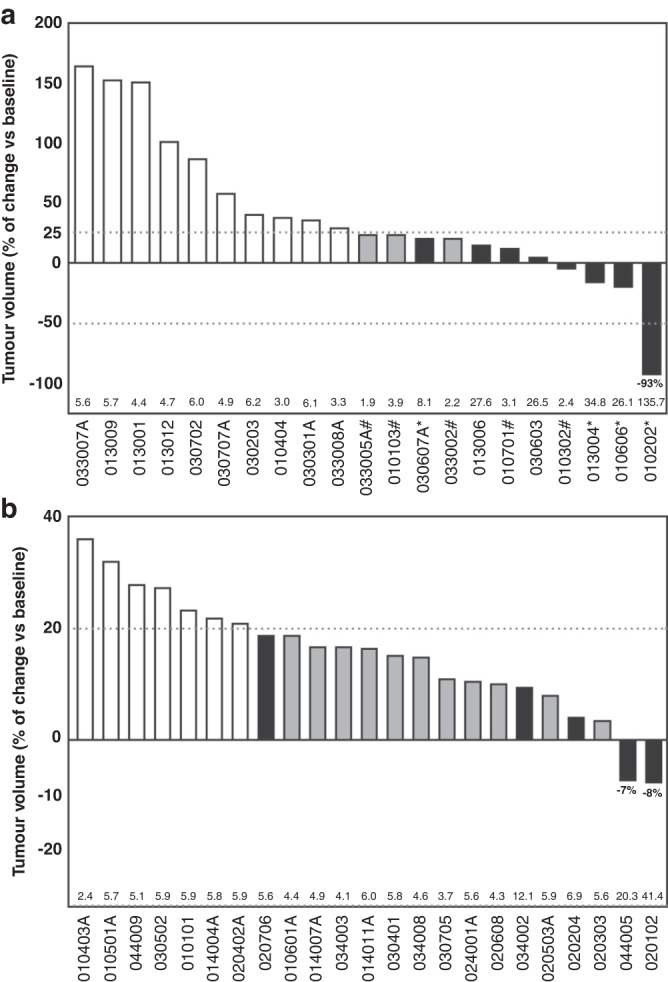
Waterfall plot responses during 2-OHOA monotherapy. Best response after initial diagnosis. **a** tumour volume of patients with glioma with respect to the baseline volume at recruitment (100%). The dotted lines describe the upper and lower limits to consider tumour progression, stable disease, and tumour regression by RANO criteria. **b** tumour volume in non-glioma patients with respect to the baseline volume (100%). In both graphs, open bars define the tumour volume in non-responding patients, grey bars are patients with stable disease or tumour regression who failed to meet all RANO or RECIST criteria and black bars are patients with stable disease or tumour regression.

**Fig. 4:**
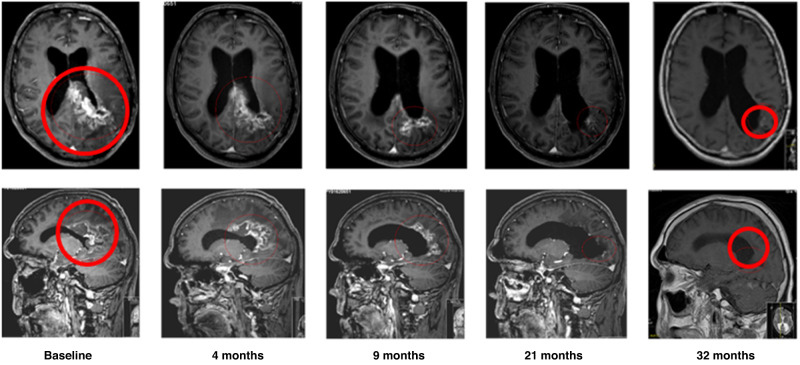
MRI scans over time of a patient with partial response. The patient showed a sustained tumour regression (partial response according to RANO criteria) over time for 3 years on monotherapy with 2-OHOA (500 mg BID, cohort 2). MRI transverse (upper scans) and sagittal (lower scans) brain images show the glioblastoma (red circle) reduction, which was determined to be ca. −93% of the initial volume.

Twenty-one HGG patients had a radiological assessment at baseline and at least one post-baseline time point, so they were included in tumour response analysis. Eight of those 21 (38.1%) HGG patients had either a partial response (PR) or stable disease (SD) by RANO criteria (Fig. 3a and Supplemental Table 3). One patient (1/21) experienced a sustained PR of >2.5 years at 1000 mg daily between cycle 1 and cycle 44, and then at 12,000 mg daily between cycle 45 and cycle 48 (Fig. 4). Seven of the 21 patients (33.3%) had at least one report of stable disease, and four of these patients achieved SD for at least 6 months. All patients had received 2 or more lines of treatment without bevacizumab. Six patients had SD at doses of 12,000 mg daily or above. Eleven (52.4%) patients derived clinical benefit with either PR or SD as their best tumour response.

Twenty-four patients with other advanced solid tumours were included in the intended-to-treat population. Although no patients achieved a PR, five of those 24 patients (20.8%; 1 mesothelioma, 1 lung metastasis of distal bile duct adenocarcinoma, 1 pancreatic adenocarcinoma, 1 metastatic lung adenocarcinoma, and 1 colon adenocarcinoma) had at least 1 assessment of SD, 3 of which lasted for 3 months or more (Fig. 3b). As all patients had demonstrated progressive disease at the time of study entry, halting tumour progression was clinically meaningful.

Therapeutic activity had been shown at low doses of 1 g/day while MTD was established at 12 g/day which is a wide therapeutic window that point out the potential of the drug and its safety profile.

A Kaplan–Meier plot of PFS in the ITT is shown in Supplemental Fig. 1, based on data shown in Supplemental Table 4. PFS was 40 days (95% CI 34.0–46.0) in glioma patients and 42 days (95% CI 39.0–48.0) in patients with other solid tumours.

## Discussion

Given the lack of effective therapies for HGG, the investigation of novel therapies is a clinical priority. 2-OHOA is a synthetic lipid able to modulate the sphingolipids metabolism that in consequence modifies the membrane composition and properties impacting on the cell signalling and avoiding cancer cell proliferation.

In this first-in-human Phase 1/2A study, 2-OHOA monotherapy was well-tolerated, with the most common AEs being diarrhoea, nausea and vomiting. Events were dose-related and anticipated given the patient population as well as the nature of the oral suspension formulation of 2-OHOA. There were no SAEs attributable to study drug or AEs resulting in death. 2-OHOA was well-tolerated at the RP2D of 4000 mg TID.

2-OHOA plasma concentrations were quantifiable across the dose range studied. Power models demonstrated dose proportionality in terms of *C*_max_ and AUC_0-8h_ over the single dose range of 250 mg to 4000 mg, and over the continuous dose range of 500 mg to 16,000 mg daily (taken as BID or TID) at steady state, and drug accumulation at higher doses was not associated with SAEs. Whilst food caused a delay in *T*_max_, this was not deemed to be clinically significant as the bioavailability of 2-OHOA was comparable in the fed and fasted state.

A limitation of this study is that tumour type heterogeneity, as well as advanced tumour stage, further hinders obtaining robust results showing the effects of lipid modulation or mRNA expression on PD, but this is being explored in subsequent studies.

2-OHOA monotherapy demonstrated promising anti-tumour activity, particularly in patients with HGG. Eight (38.1%) HGG patients experienced either a PR or SD by RANO criteria, with clinical benefit lasting at least 6 months in five patients. One patient with GBM who had been heavily pre-treated experienced a sustained partial response lasting for more than 3 years (Fig. 4). An encouraging 6-month PFS rate of 18.5% was observed in the relapsed glioma population. Activity in the other advanced solid tumours cohort was more modest, with clinical benefit lasting at least 3 months observed in three patients and a 6-month PFS rate of 3.7%.

The encouraging clinical response observed in a population with such a poor prognosis and clear unmet medical needs has supported the designation of Orphan Medicinal Product and Orphan Drug by the EMA (2011) and FDA (2021), respectively for the indication of Glioma; meriting further investigation. Three further trials have been performed. A Phase Ib trial to evaluate the safety of 2-OHOA in combination with the standard of care (chemoradiotherapy) in newly diagnosed GBM patients (NCT03867123), has completed with encouraging results to be published in 2022–2023; Second, an international pivotal Phase IIB/III study, under scientific advice by the Committee for Medicinal Products for Human Use (CHMP) of the European Medicines Agency (EMA), investigating 2-OHOA efficacy in newly diagnosed GBM adult patients is currently underway (NCT04250922) with preliminary results expected in 2023, which, if providing promising and reassuring outcomes may prove to be sufficient to support a Conditional Marketing Authorisation (CMA) following careful regulatory assessment; and third, a paediatric IB/IIA trial in USA (NCT04299191), expected to be completed in 2023.

In conclusion, monotherapy with the first-in-class orphan drug 2-OHOA has shown encouraging anti-tumour activity, particularly in HGG, in conjunction with a manageable safety profile up to very high doses; placing it as an ideal candidate for further investigation in gliomas and, potentially, other malignant diseases.

## Supplementary information

Revised Supplemental Material

## Data Availability

The data are available on the Clinical Study Report of the trial.
