# Supplementary material for: A Phase 1/2A trial of idroxioleic acid: first-in-class sphingolipid regulator and glioma cell autophagy inducer with antitumor activity in refractory glioma
Source: Br J Cancer. 2023 Jul 24;129(5):811–8. doi: 10.1038/s41416-023-02356-1 (PMC10449773; doi:10.1038/s41416-023-02356-1)
Supplement: Supplementary file 1 — Revised Supplemental Material [file 41416_2023_2356_MOESM1_ESM.docx]

**SUPPLEMENTAL MATERIAL**

**Supplemental Table 1.** **Demographic characteristics of patients, oral dose, response to treatment, and previous lines of treatment.**

|  | Age at Diagnosis | Sex | Condition | Dose  (p.o., mg/day) | Response | Previous lines of chemo (#) | Treatment:  Start Date - End Date | Pt enroll date/  PD |
| --- | --- | --- | --- | --- | --- | --- | --- | --- |
| 1 | 60 | M | MESOTHELIOMA | 500 | No | 1 | CBDCA: Feb-Mar13  Ptx: Feb-Mar13  RT: Apr-May13 | Jun13  PD  2 cycles |
| 2 | 67 | M | MESOTHELIOMA | 500 | SD^1^ | 2 | RT: 12-14 Nov 2012  Ptx+CBDCA: 22Nov12-04Jan13 | Jul13/ wd after 10m |
| 3 | 70 | F | GBM | 500 | No | 2 | RT + TMZ: Nov11-Jan12 (PD 3 cycles)  Ltine: Mar-May13 (PD 2 m) | Jul13/ PD 1Cycle |
| 4 | 19 | M | GBM | 1,000 | No | 4 | RT+TMZ: Nov11-Jan12  Bzmab: Feb-Oct12 (PD 8 m)  Bzmab+Ican: Nov12-Apr13 (PD 5m)  Bmab: May-Jun13 (PD 1 m) | Aug13/ PD 1Cycle |
| 5 | 51 | M | GBM | 1,000 | PR^2^ | 2 | RT+TMZ: Apr-Aug12 (PD 3 cycles)  Pzine+Ltine+Vcine: Nov-Feb13 (PD 4 m) | Sep13  No PD on target lesion  Wd in Jun16 |
| 6 | 65 | M | GBM | 1,000 | No | 5 | RT+TMZ: Sept-Dec2007  RT: Nov-Dec10  TMZ: Aug11-Feb12 (PD 6m)  G028070: Apr-Oct12 (CT, PD 6m)  16F-MC-JJCA: Jan-Jul13 (CT, PD 5m) | Sep13  PD  2 cycles |
| 7 | 61 | F | PANCREAS ADK | 1,000 | SD | 2 | Gem: Feb-Jul13 | Sep13  PD  2 cycles |
| 8 | 58 | M | GBM | 2,000 | No | 2 | RT+TMZ: Nov12-Jan13 (PD 2m)  Bzmab: Jan-May13 (PD 4m) | Nov13  PD  2 cycles |
| 9 | 58 | M | GBM | 2,000 | SD | 1 | RT+TMZ: May-Jul13  TMZ: Aug-Oct13 (PD 2m) | Nov13  PD  1 cycle |
| 10 | 60 | F | SMALL CELL LUNG | 2,000 | No | 4 | CBDCA: May- Jun13  Etoposide IV: May- Jun13  Etoposide Oral: May- Jun13  Topotecan: Jul-Aug13 | Dec13  PD  1 cycle |
| 11 | 63 | F | ENDOMETRIAL ADK | 4,000 | No | 5 | CBDCA: May-Sep12  Pcxel: May-Sep12  CDDP: May-Sep12  Adriamycin: May-Sep12  Tamoxifen: Feb-May13 | Feb14  PD  2 cycles |
| 12 | 50 | M | RECTAL ADK | 4,000 | No | 8 | Oxaliplatin R1: Jun-Aug12  Cap R1: Jun-Aug12  Ican R1: Dec12-April13  5-FU R2: Dec12-Apr13  OXDP: Jun13  5-FU R3: Jun13  BAL101553: Dec13  Bzmab R2: Dec12-Apr13 | Mar14  PD  2 cycles |
| 13 | 68 | F | METASTASIC RECTAL ADK | 4,000 | NA | 3 | Cap: UNK-Sep12  Xelox: UNK12-May13  5-FU+levin+IRT+Bzmad: May-Dec13 | replaced |
| 14 | 46 | F | GLIOMA | 4,000 | No | 1 | RT+TMZ: Oct-Nov13  TMZ: Dec13-Apr14 (PD 3m) | Jun14  PD  1 cycle |
| 15 | 50 | F | COLON ADK | 8,000 | No | 5 | Folfox: 08  Folfiri+cxmab:uk10-Apr11  Folfiri+cxmab: Sep11-UK  Cxmab: Apr-Sep11  Bzmab: Apr-UK13 | Aug14  PD  2 cycle |
| 16 | 44 | F | URACHAL ADK | 8,000 | No | 12 | CDDP: Aug-Oct10  5-FU: Aug-Oct10  OXDP: Apr-May12  5-FU: Apr-May12  CBDCA: Jul-Nov12  5-FU: Jan13  Fol ac: Jan13  OXDP: Jan13  Fol ac: Jan-Aug13  5-FU: Jan-Aug14  OXDP: Jan-Aug13 | Aug14  PD  2 cycle |
| 17 | 61 | M | METASTASIC SIGMOID COLON ADK | 8,000 | No | 9 | 5-FU: Apr12-Mar13  Ican: Apr12-Mar13  Fol ac: Apr12-Mar13  Bzmab: May12-Mar13  OXDP | Sep14  PD  2 cycle |
| 18 | 45 | F | METASTASIC RECTAL (KRAS WT) | 12,000 | No | 6 | Cap: UNK-Dec12  Folfox CT: UNK-Oct12  Folfori+cxmab: Mar-Sep13  Folfox : Dec13-Mar14  Folfox: Jun-Aug14  Bzmab: Jun-Aug14  RT: UNK-Dec12  RT: Dec13 | Oct2014PD  2 cycles |
| 19 | 36 | M | GBM | 12,000 | NA | 4 | TMZ: Nov11-Jan12  TMZ: Feb-Jul12  TMZ: Apr-May14  IRT: Aug-Sep14  Bzmab: Aug-Sep1  RT: Nov11-Jan12 | replaced |
| 20 | 63 | F | GBM | 12,000 | SD | 2 | RT+TMZ: Jul-Sep13  TMZ: Oct13-Mar14 (PD 5m)  TMZ: Jul-Oct14 (PD 3m) | Dec14  PD  1 cycle |
| 21 | 66 | M | COLON ADK | 12,000 | No | 11 | Cap: UNK-UNK05  OXPD: UNK-UNK05  IRT: Jan-Apr08  Cxmab: Jan-Apr08  Cap: Nov-UNK12  IRT: Nov-UNK12  Fol ac: Jan13  5-FU: Jan13  IRT:Jan13  IRT: Mar13  Cap:Mar13  RT: Mar-Mai11  RT:Aug-Sep13 | Dec14  PD  1 cycle |
| 22 | 65 | M | OLIGOASTROCYTOMA | 12,000 | NA | 2 | TMZ: Oct13-Feb14  Bzmab: Feb-Dec14  RT: Oct-Dec13 | replaced |
| 23 | 57 | M | GBM | 12,000 | SD | 2 | RT+TMZ: May-Jun13  TMZ: Jul-Dec13 (PD 6m)  PCV: May-Nov14 (PD 6m) | Feb15  No PD  Wd new lesion |
| 24 | 67 | M | ASTROCYTOMA | 12,000 | SD | 3 | RT: Mar-May13  TMZ: May-Nov13 (PD 5m)  Ican+Bzmab: Jul14-Jan15 (PD 6m) | Feb15  PD  2 cycle |
| 25 | 76 | M | METASTASIC RECTO-SIGMOID JUNCTION ADK | 12,000 | No | 12 | IRT: Jan-Jun09  Fol ac: Jan-Jun09  5-FU: Jan-Jul09  IRT: Oct10-Jan11  Fol ac: Oct10-Jan11  5-FU: Oct10-Jan11  IRT: Feb-Apr11  Cap: Feb-Apr11  Bzmab: Dec11-Dec13  OXPD: Dec11-May12  Cap: Dec11-Oct12  Czmib: May-Sep14 | Feb15  PD  2 cycle |
| 26 | 39 | F | G3 GLIOMA | 16,000 | SD | 2 | RT:09  PVC: Oct12-Jan13  TMZ: Jul-Sp14 (PD 2m) | Apr15  No PD  Wd |
| 27 | 57 | F | ASTROCYTOMA | 16,000 | No | 2 | RT+TMZ: Apr-May12  TMZ: Jun-Nov12  TMZ: Mar-Apr14 | Apr15  PD  2 cycle |
| 28 | 73 | M | PLEURAL MESOTHELIOMA | 16,000 | NA | 4 | CBDCA: Jun-Aug14  Ptx: Jun-Aug14  GSK2256098: Nov14-Jan15  Trnib: Nov14-Jan15  RT: Sep14 | replaced |
| 29 | 65 | F | CHONDROSARCOMA OF THE UTERUS | 16,000 | No | 4 | DOX: Oct-Dec12  Imide: Feb-Apr14  Pnib: Jun14-Mar15  Trdin: Apr-May15 | Jul15  PD  1 cycle |
| 30 | 50 | F | ENDOMETRIAL ADK | 16,000 | No | 7 | CBCDA: Nov12-Mar13  Taxol: Nov12-Mar13  MA: Aug-Oct13  AZ108 CT: Feb-Aug14  MLN1117 CT: Feb15  CBCDA: Apr-May15  Pcxel: Apr-May15 | Aug15  PD  3 cycle |
| 31 | 71 | M | METASTASIC LUNG ADK | 16,000 | SD | 3 | CBCDA: Feb-Mar14  Ptx: Feb-Mar14  Dcxel: Sep-Nov14  RT: Aug14 | Aug15  PD  3 cycle |
| 32 | 25 | M | ANAPLASTIC ASTROCYTOMA | 16,000 | No | 4 | RT: Mar14  TMZ: Apr-Oct14  Ican: Jul15  Bzmab: Jul-Sep15 | Oct15  PD  2 cycles |
| 33 | 42 | F | GBM | 12,000 | No | 3 | RT+TMZ: Mar-Apr14  TMZ: May-Jul14  PVC: Aug-Mar15  CCNU: Apr-Sep15 | Dec15  PD  <1 cycle |
| 34 | 52 | M | GBM | 12,000 | No | 1 | RT: Jun-Jul15  TMZ: Aug-Oct15 | Dec15  Wd |
| 35 | 57 | M | GLIOMA | 12,000 | NA | 1 |  |  |
| 36 | 38 | M | OLIGODENDROGLIOMA | 12,000 | SD | 2 | PCV: Feb-Sep11  PCV: Mar-Jul14  RT+TMZ: Feb-Mar14  TMZ: Apr-Sep14 | Dec15  PD  9 cycles |
| 37 | 68 | M | GBM | 12,000 | No | 4 | RT: Jan-Apr13  TMZ: Apr-Oct13  TMZ: Jan-Jul15  Fine: Aug-Sep15  Bzmab: Oct15 | Dec15  PD  < 1m |
| 38 | 48 | M | GBM | 12,000 | SD | 2 | RT+TMZ: Apr-May15  PCV: Jul-Nov15 | Jan16  PD  7cycles |
| 39 | 56 | M | GBM | 12,000 | No | 6 | RT+TMZ: May-Jun13  BKM120: May13  TMZ: Jul-Dec13  Gliadel: Oct14  Bzmab: Feb-Nov15  Ltine: Feb-Nov15 | Jan16  PD  1m |
| 40 | 49 | M | GLIOSARCOMA | 12,000 | No | 6 | RT+TMZ: Aug-Oct13  TMZ: Oct13-Apr14  Bzmab: Oct13-Mar15  Ltine+Bzmab: Apr15 (CT)  Ican+Bzmab: May15 (CT)  CBDCA: Oct-Dec15 | Jan16  PD  1 cycle |
| 41 | 53 | F | GLIOMATOSIS CEREBRI | 12,000 | No | 2 | RT+TMZ: Jul14  TMZ: Sep14-Aug15  PVC: Oct-Dec15 | Feb16  PD  1 cycle |
| 42 | 63 | F | GBM | 12,000 | NA | 2 | TMZ: Oct-Nov14  TMZ: Oct15  RT: Oct-Nov14 | replaced |
| 43 | 50 | M | GBM | 12,000 | NA | 5 | TMZ: Mar-Oct14  IRT: Dec14-Sep15  Bzmab: Dec14-Sep15  Fine: Oct-Nov11  TMZ: Dec15-Feb16  RT: Mar-Apr14 | replaced |
| 44 | 50 | F | GBM | 12,000 | No | 1 | RT+TMZ: Mar-Apr15 | Apr16  PD  1 cycle |
| 45 | 75 | M | METASTASIC COLORECTAL ADK | 12,000 | No | 4 | 5-FU: Nov04-Jul05  Cap: Aug-Sep13  Bzmab: Aug13  Rtxed: Nov11-Aug14  RT: Oct-Nov15 | Dec15  PD  3 cycle |
| 46 | 75 | F | COLON ADK | 12,000 | SD | 7 | Fol ac.+5-FU+Oxpt: Feb-uk13  Cxmab: Feb13-Feb14  Fol ac. +5-FU+Icam: Fb14-Nov15 | Dec15  Wd  new lesion |
| 47 | 73 | M | RECTUM ADK | 12,000 | No | 8 | Fol ac: Dec14-May15  5-FU: Dec14-May15  IRT: Dec14-May15  Fol ac: May-Aug15  5-FU: May-Aug15  OXDP: May-Aug15  R06895882: Oct-Nov15  Ozmab: Oct15  RT: Jun15 | Dec15  PD  2 cycle |
| 48 | 68 | F | COLORECTAL CARCINOMA | 12,000 | No | 6 | Folfox: Nov10-Jun11  Bzmab: Nov10-Jun11  IRT: UNK/UNK-Jul14  Abrept: UNK/UNK-Jul14  Folfox: Nov14-Mar15  Rfnib: Jul-Nov15 | Jan16  PD  2 cycle |
| 49 | 55 | M | DISTAL BILE DUCT ADK | 12,000 | SD | 3 | Ptx: Aug-Sep15  Nmab: Oct-Dec15  CDDP: Aug-Sep15 | Jan16  Wd new lesion |
| 50 | 78 | M | METASTASIC SCC OF OESOPHAGUS | 12,000 | NA | 3 | Ebcin: Dec14-Feb15  OXDP: Dec14-Jun15  Bzmab: Dec14-Jun15  RT: Oct15 | replaced |
| 51 | 74 | M | METASTASIC RECTAL ADK | 12,000 | No | 6 | Cap+OXDP: May-Jul12  Bzmab: Sept-Oct13  IRT+ 5-FU: Sep-Nov13  IRT+5FU+Fol ac: UNK13-UNK14  IRT+5FU+Fol ac: Jun14-May15  Folfox: Sept-Oct15  RT: Jun-Jul12 | Feb16  PD  2 cycle |
| 52 | 67 | F | RECTUM | 12,000 | No | 14 | Fol ac: Jul-Oct13  5-FU: Jul-Oct13  OXDP: Jul-Oct13  5-FU: Oct13-Mar14  IRT: Oct13-Mar14  Cxmab: OCt13-Mar14  5-FU: Aug14-May15  IRT: Aug14-May15  Cxmab: Aug14-May15  5-FU: Apr-Jun15  Fol ac: Jun-Nov15  5-FU: Jun-Nov15  OXPD: Jun-Nov15  Abrecpt: Jun-Nov15  RT_Apr-Jun14 | Feb16  PD  3 cycle |
| 53 | 67 | F | GALLBLADDER ADK | 12,000 | No | 4 | Gem: Jan-Jun14  Cap: Dec14-May15  Gem+CDDP: Jun-Sep15  Aktinh: Dec15-Jan16 | Feb16  PD  3 cycle |
| 54 | 74 | M | CAECUM ADK | 12,000 | No | 5 | Folfiri: Jun-Oct15  Rfnib: Nov15-Jan16  OXPD: Nov14-Mar15  Cap: Nov14-Mar15  Bzmab: Nov14-Mar15 | Mar16  PD  2 cycle |
|  | 57.6 yr | 32M/22F |  |  |  |  |  |  |

Abbreviations: NA=not applicable, PR=partial response, SD=stable disease, wd=withdrawal, pt=patient

**Supplemental Table 2.** **Molecular Markers of glioma patients**

|  | Age at Diagnosis | Sex | Condition | Molecular Specification | |
| --- | --- | --- | --- | --- | --- |
|  |  |  |  | Markers  Malignant Glioma | Result |
| 3 | 70 | F | GBM | MGMT promoter methylation status | UNK |
|  |  |  |  | 1p19 co-deletion | UNK |
|  |  |  |  | IDH 1/2 mutation | wt |
|  |  |  |  | BRAF mutation | No |
|  |  |  |  | PTEN mutation | Yes |
|  |  |  |  | EGFR amplification | No |
|  |  |  |  | EGFR mutation | No |
|  |  |  |  | MAPK13 alteration | NA |
|  |  |  |  | KRAS alteration | No |
|  |  |  |  | MET (N3755) | Yes |
|  |  |  |  | PIK3CA (R115L) | Yes |
|  |  |  |  | PIK3CA (E542K) | Yes |
|  |  |  |  | TP53 (K132N) | Yes |

| 4 | 19 | M | GBM | MGMT promoter methylation status | UNK |
| --- | --- | --- | --- | --- | --- |
|  |  |  |  | 1p19 co-deletion | UNK |
|  |  |  |  | IDH 1/2 mutation | UNK |
|  |  |  |  | BRAF mutation | UNK |
|  |  |  |  | PTEN mutation | UNK |
|  |  |  |  | EGFR amplification | UNK |
|  |  |  |  | EGFR mutation | UNK |
|  |  |  |  | MAPK13 alteration | NA |
|  |  |  |  | KRAS alteration | NA |
| 5 | 51 | M | GBM | MGMT promoter methylation status | UNK |
|  |  |  |  | 1p19 co-deletion | UNK |
|  |  |  |  | IDH 1/2 mutation | UNK |
|  |  |  |  | BRAF mutation | No |
|  |  |  |  | PTEN mutation | No |
|  |  |  |  | EGFR amplification | No |
|  |  |  |  | EGFR mutation | No |
|  |  |  |  | MAPK13 alteration | NA |
|  |  |  |  | KRAS alteration | NA |
|  |  |  |  | ATM (T17691), | Yes |
|  |  |  |  | FBXW7 (R484K) | Yes |
| 6 | 65 | M | GBM | MGMT promoter methylation status | UNK |
|  |  |  |  | 1p19 co-deletion | UNK |
|  |  |  |  | IDH 1/2 mutation | UNK |
|  |  |  |  | BRAF mutation | UNK |
|  |  |  |  | PTEN mutation | UNK |
|  |  |  |  | EGFR amplification | UNK |
|  |  |  |  | EGFR mutation | UNK |
|  |  |  |  | MAPK13 alteration | NA |
|  |  |  |  | KRAS alteration | NA |
| 8 | 58 | M | GBM | MGMT promoter methylation status | Un- |
|  |  |  |  | 1p19 co-deletion | UNK |
|  |  |  |  | IDH 1/2 mutation | UNK |
|  |  |  |  | BRAF mutation | UNK |
|  |  |  |  | PTEN mutation | UNK |
|  |  |  |  | EGFR amplification | UNK |
|  |  |  |  | EGFR mutation | UNK |
|  |  |  |  | MAPK13 alteration | NA |
|  |  |  |  | KRAS alteration | NA |
| 9 | 58 | M | GBM | MGMT promoter methylation status | UNK |
|  |  |  |  | 1p19 co-deletion | UNK |
|  |  |  |  | IDH 1/2 mutation | wt |
|  |  |  |  | BRAF mutation | No |
|  |  |  |  | PTEN mutation | Yes |
|  |  |  |  | EGFR amplification | No |
|  |  |  |  | EGFR mutation | No |
|  |  |  |  | MAPK13 alteration | NA |
|  |  |  |  | KRAS alteration | No |
| 14 | 46 | F | Glioma | MGMT promoter methylation status | UNK |
|  |  |  |  | 1p19 co-deletion | UNK |
|  |  |  |  | IDH 1/2 mutation | wt |
|  |  |  |  | BRAF mutation | No |
|  |  |  |  | PTEN mutation | Yes |
|  |  |  |  | EGFR amplification | No |
|  |  |  |  | EGFR mutation | No |
|  |  |  |  | MAPK13 alteration | NA |
|  |  |  |  | KRAS alteration | No |
| 19 | 36 | M | GBM | MGMT promoter methylation status | UNK |
|  |  |  |  | 1p19 co-deletion | UNK |
|  |  |  |  | IDH 1/2 mutation | UNK |
|  |  |  |  | BRAF mutation | No |
|  |  |  |  | PTEN mutation | UNK |
|  |  |  |  | EGFR amplification | UNK |
|  |  |  |  | EGFR mutation | UNK |
|  |  |  |  | MAPK13 alteration | NA |
|  |  |  |  | KRAS alteration | NA |
| 20 | 63 | F | GBM | MGMT promoter methylation status | UNK |
|  |  |  |  | 1p19 co-deletion | UNK |
|  |  |  |  | IDH 1/2 mutation | UNK |
|  |  |  |  | BRAF mutation | UNK |
|  |  |  |  | PTEN mutation | No |
|  |  |  |  | EGFR amplification | UNK |
|  |  |  |  | EGFR mutation | No |
|  |  |  |  | MAPK13 alteration | NA |
|  |  |  |  | KRAS alteration | NA |
| 22 | 65 | M | Oligoastro-cytoma (glioma) | MGMT promoter methylation status | UNK |
|  |  |  |  | 1p19 co-deletion | UNK |
|  |  |  |  | IDH 1/2 mutation | wt |
|  |  |  |  | BRAF mutation | UNK |
|  |  |  |  | PTEN mutation | UNK |
|  |  |  |  | EGFR amplification | UNK |
|  |  |  |  | EGFR mutation | UNK |
|  |  |  |  | MAPK13 alteration | NA |
|  |  |  |  | KRAS alteration | NA |
|  |  |  |  | P53 amplification | Yes |
| 23 | 57 | M | GBM | MGMT promoter methylation status | UNK |
|  |  |  |  | 1p19 co-deletion | UNK |
|  |  |  |  | IDH 1/2 mutation | UNK |
|  |  |  |  | BRAF mutation | UNK |
|  |  |  |  | PTEN mutation | UNK |
|  |  |  |  | EGFR amplification | UNK |
|  |  |  |  | EGFR mutation | UNK |
|  |  |  |  | MAPK13 alteration | NA |
|  |  |  |  | KRAS alteration | NA |
| 24 | 67 | M | Astrocytoma | MGMT promoter methylation status | UNK |
|  |  |  |  | 1p19 co-deletion | UNK |
|  |  |  |  | IDH 1/2 mutation | UNK |
|  |  |  |  | BRAF mutation | UNK |
|  |  |  |  | PTEN mutation | UNK |
|  |  |  |  | EGFR amplification | UNK |
|  |  |  |  | EGFR mutation | UNK |
|  |  |  |  | MAPK13 alteration | NA |
|  |  |  |  | KRAS alteration | NA |
|  |  |  |  | PGFA mutation, | Yes |
|  |  |  |  | P53 mutation | Yes |
|  |  |  |  | WT-1 mutation | Yes |
| 26 | 39 | F | G3 glioma | MGMT promoter methylation status | Met |
|  |  |  |  | 1p19 co-deletion | No |
|  |  |  |  | IDH 1/2 mutation | Mut |
|  |  |  |  | BRAF mutation | No |
|  |  |  |  | PTEN mutation | No |
|  |  |  |  | EGFR amplification | No |
|  |  |  |  | EGFR mutation | No |
|  |  |  |  | MAPK13 alteration | No |
|  |  |  |  | KRAS alteration | No |
| 27 | 57 | F | Astrocytoma | MGMT promoter methylation status | UNK |
|  |  |  |  | 1p19 co-deletion | UNK |
|  |  |  |  | IDH 1/2 mutation | UNK |
|  |  |  |  | BRAF mutation | No |
|  |  |  |  | PTEN mutation | No |
|  |  |  |  | EGFR amplification | No |
|  |  |  |  | EGFR mutation | No |
|  |  |  |  | MAPK13 alteration | No |
|  |  |  |  | KRAS alteration | No |
| 32 | 25 | M | Anaplastic Astrocytoma | MGMT promoter methylation status | Met |
|  |  |  |  | 1p19 co-deletion | UNK |
|  |  |  |  | IDH 1/2 mutation | wt |
|  |  |  |  | BRAF mutation | UNK |
|  |  |  |  | PTEN mutation | UNK |
|  |  |  |  | EGFR amplification | UNK |
|  |  |  |  | EGFR mutation | UNK |
|  |  |  |  | MAPK13 alteration | NA |
|  |  |  |  | KRAS alteration | NA |
|  |  |  |  | P53 amplification | Yes |
| 33 | 42 | F | GBM | MGMT promoter methylation status | Un- |
|  |  |  |  | 1p19 co-deletion | No |
|  |  |  |  | IDH 1/2 mutation | wt |
|  |  |  |  | BRAF mutation | No |
|  |  |  |  | PTEN mutation | No |
|  |  |  |  | EGFR amplification | No |
|  |  |  |  | EGFR mutation | No |
|  |  |  |  | MAPK13 alteration | NA |
|  |  |  |  | KRAS alteration | NA |
| 34 | 52 | F | GBM | MGMT promoter methylation status | UNK |
|  |  |  |  | 1p19 co-deletion | UNK |
|  |  |  |  | IDH 1/2 mutation | wt |
|  |  |  |  | BRAF mutation | UNK |
|  |  |  |  | PTEN mutation | UNK |
|  |  |  |  | EGFR amplification | Yes |
|  |  |  |  | EGFR mutation | UNK |
|  |  |  |  | MAPK13 alteration | NA |
|  |  |  |  | KRAS alteration | NA |
| 35 | 57 | M | Glioma | MGMT promoter methylation status | UNK |
|  |  |  |  | 1p19 co-deletion | UNK |
|  |  |  |  | IDH 1/2 mutation | UNK |
|  |  |  |  | BRAF mutation | UNK |
|  |  |  |  | PTEN mutation | UNK |
|  |  |  |  | EGFR amplification | UNK |
|  |  |  |  | EGFR mutation | UNK |
|  |  |  |  | MAPK13 alteration | NA |
|  |  |  |  | KRAS alteration | NA |
| 36 | 38 | M | Oligodendro-glioma | MGMT promoter methylation status | UNK |
|  |  |  |  | 1p19 co-deletion | Yes |
|  |  |  |  | IDH 1/2 mutation | UNK |
|  |  |  |  | BRAF mutation | UNK |
|  |  |  |  | PTEN mutation | No |
|  |  |  |  | EGFR amplification | UNK |
|  |  |  |  | EGFR mutation | UNK |
|  |  |  |  | MAPK13 alteration | NA |
|  |  |  |  | KRAS alteration | NA |
| 37 | 68 | M | GBM | MGMT promoter methylation status | Un- |
|  |  |  |  | 1p19 co-deletion | UNK |
|  |  |  |  | IDH 1/2 mutation | UNK |
|  |  |  |  | BRAF mutation | UNK |
|  |  |  |  | PTEN mutation | UNK |
|  |  |  |  | EGFR amplification | UNK |
|  |  |  |  | EGFR mutation | UNK |
|  |  |  |  | MAPK13 alteration | NA |
|  |  |  |  | KRAS alteration | NA |
| 38 | 48 | M | GBM | MGMT promoter methylation status | Un- |
|  |  |  |  | 1p19 co-deletion | No |
|  |  |  |  | IDH 1/2 mutation | wt |
|  |  |  |  | BRAF mutation | No |
|  |  |  |  | PTEN mutation | Yes |
|  |  |  |  | EGFR amplification | No |
|  |  |  |  | EGFR mutation | No |
|  |  |  |  | MAPK13 alteration | NA |
|  |  |  |  | KRAS alteration | No |
|  |  |  |  | BLM (K323R) | Yes |
|  |  |  |  | RAD51B (Y180C) | Yes |
| 39 | 56 | F | GBM | MGMT promoter methylation status | UNK |
|  |  |  |  | 1p19 co-deletion | UNK |
|  |  |  |  | IDH 1/2 mutation | wt |
|  |  |  |  | BRAF mutation | UNK |
|  |  |  |  | PTEN mutation | Yes |
|  |  |  |  | EGFR amplification | UNK |
|  |  |  |  | EGFR mutation | UNK |
|  |  |  |  | MAPK13 alteration | NA |
|  |  |  |  | KRAS alteration | NA |
| 40 | 49 | M | Gliosarcoma | MGMT promoter methylation status | UNK |
|  |  |  |  | 1p19 co-deletion | UNK |
|  |  |  |  | IDH 1/2 mutation | UNK |
|  |  |  |  | BRAF mutation | UNK |
|  |  |  |  | PTEN mutation | UNK |
|  |  |  |  | EGFR amplification | Yes |
|  |  |  |  | EGFR mutation | Yes |
|  |  |  |  | MAPK13 alteration | NA |
|  |  |  |  | KRAS alteration | NA |
| 41 | 53 | F | Gliomatosis cerebri | MGMT promoter methylation status | UNK |
|  |  |  |  | 1p19 co-deletion | UNK |
|  |  |  |  | IDH 1/2 mutation | UNK |
|  |  |  |  | BRAF mutation | UNK |
|  |  |  |  | PTEN mutation | UNK |
|  |  |  |  | EGFR amplification | UNK |
|  |  |  |  | EGFR mutation | UNK |
|  |  |  |  | MAPK13 alteration | NA |
|  |  |  |  | KRAS alteration | NA |
| 42 | 63 | F | GBM | MGMT promoter methylation status | UNK |
|  |  |  |  | 1p19 co-deletion | UNK |
|  |  |  |  | IDH 1/2 mutation | wt |
|  |  |  |  | BRAF mutation | UNK |
|  |  |  |  | PTEN mutation | UNK |
|  |  |  |  | EGFR amplification | UNK |
|  |  |  |  | EGFR mutation | UNK |
|  |  |  |  | MAPK13 alteration | NA |
|  |  |  |  | KRAS alteration | NA |
| 43 | 50 | M | GBM | MGMT promoter methylation status | UNK |
|  |  |  |  | 1p19 co-deletion | UNK |
|  |  |  |  | IDH 1/2 mutation | wt |
|  |  |  |  | BRAF mutation | UNK |
|  |  |  |  | PTEN mutation | UNK |
|  |  |  |  | EGFR amplification | UNK |
|  |  |  |  | EGFR mutation | UNK |
|  |  |  |  | MAPK13 alteration | NA |
|  |  |  |  | KRAS alteration | NA |
| 44 | 50 | F | GBM | MGMT promoter methylation status | Un- |
|  |  |  |  | 1p19 co-deletion | No |
|  |  |  |  | IDH 1/2 mutation | UNK |
|  |  |  |  | BRAF mutation | No |
|  |  |  |  | PTEN mutation | No |
|  |  |  |  | EGFR amplification | No |
|  |  |  |  | EGFR mutation | No |
|  |  |  |  | MAPK13 alteration | NA |
|  |  |  |  | KRAS alteration | No |
|  |  |  |  | ATRX (1360fs*6) | Yes |
|  |  |  |  | RB1 (E926G) | Yes |
|  |  |  |  | RET (M1109T) | Yes |
|  |  |  |  | TP53 (C1765;R273C) | Yes |

Abreviations: UNK: unknow; NA: No available; Wt: wildtype; Un-: unmethylated; Met: metilated; Mut: mutated

**Supplemental Table 3. Best overall response and duration of response in glioma patients with stable disease or partial response by RANO criteria**

| Treatment Cohort (per day) | Patient Age (years) | Diagnosis | Treatment  Duration | Best Overall Response | Estimated Duration of Response |
| --- | --- | --- | --- | --- | --- |
| 1,000mg | 51 | Glioblastoma | C48D8 | PR | 33 months |
| 2,000mg | 58 | Glioblastoma | C1D15 | SD | 1 month |
| 12,000mg (dose escalation) | 63 | Glioblastoma | C9D8 | SD | 6 months |
|  | 57 | Glioblastoma | C9D8 | SD | 6 months |
|  | 67 | Astrocytoma | C4D1 | SD | 2 months |
| 16,000mg | 39 | Grade 3 glioma | C2D1 | SD | 1 month |
| 12,000mg (glioma expanded safety cohort) | 68 | Glioblastoma | C1D8 | SD | 8 months |
| 12,000mg (glioma expanded safety cohort) | 56 | Glioblastoma | C2D8 | SD | 7 months |
| Clinical benefit rate | | | | PR or SD>6months | 5/21 (23.8%) |
|  |  |  |  | PR or SD < 6 months | 8/21 (38.1%) |

Abbreviations: PR=partial response, SD=stable disease

**Supplemental Table 4.**

| **Tumour Type** | **Study Population** | **PFS Overall** | | | **PFS at 6 months**  **N (%)** | **PFS <6months**  **N (%)** |
| --- | --- | --- | --- | --- | --- | --- |
|  |  | **Evaluable**  **N (%)** | **Censored**  **N (%)** | **Median PFS (days)**  **(95%CI)** |  |  |
| **Glioma** | ITT (N=27) | 24 (88.9%) | 3 (11.1%) | 40 (34.0-46.0) | 5 (18.5%) | 19 (81.5%) |
|  | PPS (N=14) | 14 (100%) | 0 | 44 (39.0-188.0) | 5 (35.7%) | 9 (64.3%) |
| **Other solid tumours** | ITT (N=27) | 23 (85.2%) | 4 (19.2%) | 42 (39.0-48.0) | 1 (3.7%) | 22 (96.3%) |
|  | PPS (N=19) | 18 (94.7%) | 1 (5.3%) | 42 (36.0-49.0) | 1 (5.3%) | 17 (94.7%) |

Abbreviations: PFS=progression free survival, ITT=intention to treat, PPT=per protocol

**Supplemental Figure 1. Kaplan-Meier Plot of Progression Free Survival (ITT Population).** Data from Supplemental table 4.


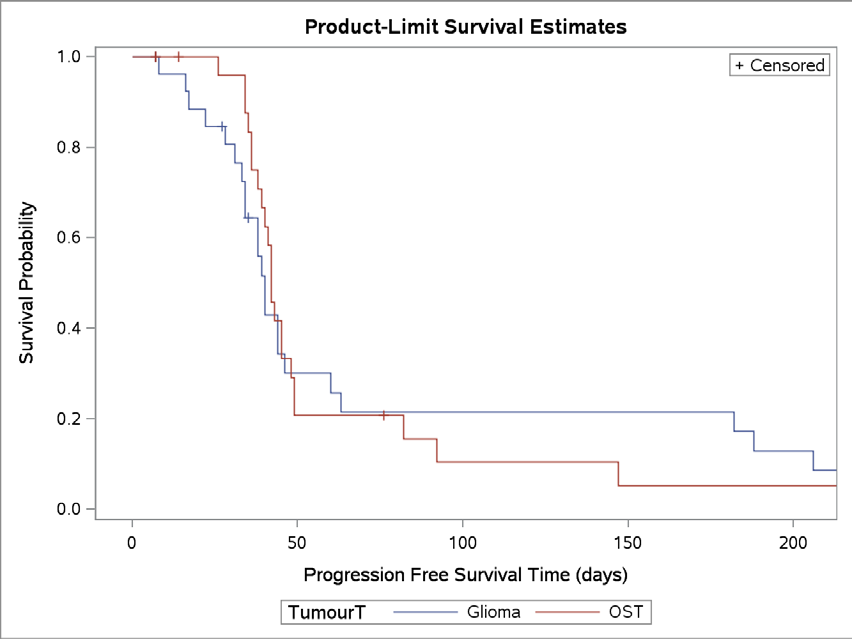


**Supplemental text 1.** Full eligibility criteria

**Inclusion Criteria**

- Able and willing to give written informed consent
- Male or female patients ≥18 years of age
- With histologically- or cytologically-confirmed advanced solid malignancy that is refractory to standard-of-care treatment, or for which there is no standard therapy
- If this is glioma:
- Grade III / Grade IV malignant glioma recurring or progressing after first or second line standard of care treatment and
- True progressive disease, confirmed according to the RANO criteria
- A life-expectancy of at least 12 weeks
- ECOG performance status of 0–2
- Able to swallow and ingest oral medication
- Able to undergo adequate tumor imaging, via CT or MRI scans, to evaluate disease evolution
- Availability of paraffin-embedded archival material for possible genomic evaluation
- Hematology values at screening/baseline: hemoglobin ≥90 g/L (9 g/dL) or 5.6 mmol/L, absolute neutrophil count ≥1.5 x 10^9^/L, platelets ≥100 x 10^9^/L
- Coagulation values at screening/baseline: International Normalized Ratio (INR) ≤1.5, partial thromboplastin time (PTT) ≤2 x upper limit of normal (ULN)
- Liver function test values at screening/baseline: total bilirubin ≤1.5 × ULN – unless explained by a genetic syndrome such as Gilberts; alanine aminotransferase (ALT) and aspartate aminotransferase (AST) ≤2.5 × ULN
- Renal function test value at screening/baseline: serum creatinine ≤1.5 x ULN
- No history of corrected QT interval (QTc) prolongation, and a normal QTc interval at screening/baseline (QTc ≤450 msec)
- Female patients (or male patient whose partner is) of non-childbearing potential (defined as >2 years after last menstruation or surgically sterile), female patients of childbearing potential with a negative serum pregnancy test within 7 days prior to the first dose of 2‑OHOA, or within 14 days followed by a confirmatory negative urine pregnancy test within 7 days prior to first dose of 2-OHOA, and using (or if male and not surgically sterile, whose partner is using) effective, non-hormonal means of contraception (non-hormonal intrauterine contraceptive device, barrier method of contraception in conjunction with spermicidal gel)

For patients with solid tumors other than glioma:

- The presence of lesions suitable for biopsy (mandatory for non-glioma patients enrolled in the expanded safety cohort and highly desirable for non-glioma patients enrolled in the dose escalation phase)

**Exclusion Criteria**

- Known hypersensitivity to any component of the study drug
- Use of any other investigational drug in the 30 days prior to the first dose of 2-OHOA
- Anti-cancer therapy within 4 weeks prior to the first dose of 2-OHOA (6 weeks for mitomycin and nitrosureas and 2 weeks for palliative radiotherapy)
- Any NCI CTCAE >Grade 1 toxicities from prior chemotherapy or radiotherapy that could impact on safety outcome assessment
- Any surgery within 14 days prior to the first dose of 2-OHOA
- Known recent >Grade 1 intracranial or intratumoral hemorrhage either by CT or MRI scan. Patients with resolving hemorrhage changes, punctuate hemorrhage or hemosiderin may enter the study
- Significant or uncontrolled cardiovascular disease, including New York Heart Association Class 3-4 heart failure, a left ventricular ejection fraction which is clinically significantly abnormal as measured by 2 dimensional (2D) echocardiogram or Multi Gated Acquisition (MUGA) scan, unstable angina or myocardial infarction within the preceding 6 months
- Known impairment of gastrointestinal function that could alter the absorption of study drug (e.g. active Crohn’s disease, malabsorption syndrome or states, unresolved diarrhea, small bowel resection or gastric by-pass surgery)
- A history of uncontrolled hyperlipidemia and/or the need for concurrent lipid-lowering therapy
- Concurrent severe and/or uncontrolled other medical diseases (e.g. uncontrolled diabetes mellitus, active uncontrolled infection) that could compromise participation in the study
- Need for warfarin, phenytoin or sulphonylureas (glibenclamide, glimepiride, glipizide, glyburide or nateglanide)
- Females who are pregnant or breastfeeding
- Any serious and/or unstable pre-existing medical, psychiatric or other condition which in the Investigator’s opinion could interfere with subject safety, obtaining written informed consent, or compliance with the study protocol

**Supplemental text 2.** Dose escalation and Dose Limiting Toxicities (DLTs) criteria and definitions.

A DLT was defined by the occurrence of any of the following toxicities defined in the National Cancer (NCI) Common Terminology Criteria for AEs (CTCAE) Version 4.03 and which were considered by the Investigator to be related to 2‑OHOA:

- ≥Grade 3 febrile neutropenia (absolute neutrophil count <1.0 x 10^9^/L with a sustained temperature of ≥38ºC for more than 1 hour) or Grade 4 neutropenia (absolute neutrophil count <0.5 x 10^9^/L) for more than 7 consecutive days
- ≥Grade 3 thrombocytopenia (platelet count 25 to <50 x 10^9^/L) with bleeding or Grade 4 thrombocytopenia (platelet count <25 x 10^9^/L)
- ≥Grade 3 non-hematological toxicity of any duration, *except:*
- Grade 3 or 4 nausea/vomiting or diarrhea was considered a DLT only if it persisted despite optimal medical management
- Alopecia was not considered a DLT
- Reversible temporary laboratory changes without clinical symptoms or relevance (discussed individually upon occurrence by the active Investigators and the Medical Monitor)
- Any other toxicity occurring at any time during the study that in the view of the active Investigators and the Medical Monitor represented a clinically significant hazard to the patient
- Any other toxicity that prevented the patient from taking at least 80% of doses during cycle 1
